# Extensive psoriasis induced by pegylated interferon: a case report

**DOI:** 10.1186/1752-1947-1-86

**Published:** 2007-09-17

**Authors:** Vincenzo Citro, Raffaele Fristachi, Giovanni Tarantino

**Affiliations:** 1 U.O.C. of General Medicine, Hepatological Unit, "Mauro Scarlato" Hospital, Scafati (SA), Italy; 2 U.O.C. of Pathology, Ospedali Riuniti delle Tre Valli, ASL SA/1 Nocera Inferiore, Italy; 3 Department of Clinical and Experimental Medicine, Federico II University Medical School of Naples, Italy

## Abstract

This paper describes the clinical course of a patient with chronic hepatitis C, genotype 2a/2c, previously treated with Interferon α2b and subsequently with Lymphoblastoid Interferon without any response, and also without any cutaneous side effects. The patient, a 50 year-old woman, was re-treated with Pegylated α2b Interferon plus Ribavirin for 24 weeks, at standard doses; during the third month of therapy she developed a mild form of psoriasis. However, encouraged by the progressive improvement of her transaminase levels and viral load decrease, the patient asked to continue the treatment; she normalized the transaminase levels during the fourth month and showed HCV-RNA negativity during the fifth month of therapy. Nevertheless, the psoriasis become worse, extending to over 75% of her body. Therapy was completed after sixth months. A month after the therapy was ceased, the patient's psoriasis receded spontaneously and completely. During the subsequent four years the patient did not experience any recurrence of either the hepatic disease or the psoriasis.

## Background

In patients suffering from chronic hepatitis C Interferon (IFN) therapy can induce various side effects, especially of autoimmune type; of these, thyroiditis, thrombocytopenia, systemic lupus erythematosus and rheumatoid arthritis are the most frequent. A certain susceptibility for immunologic abnormalities [1] plays a key role. Further more, side effects can also occur involving the skin including vasculitis, necrosis, ulceration, and alopecia [2,3]. Exacerbation of pre-existent psoriasis [3-6] and induction of psoriasis have also been described [7].

## Case presentation

A 50 year-old woman with HCV-related chronic hepatitis, without history of psoriasis, had been previously treated with 2 cycles of IFN: firstly she had received recombinant IFN alpha α2b (Intron A^®^, Schering-Plough) 3 MU trice/week for 36 weeks (September 1996–May 1997); then Lymphoblastoid IFN (Wellferon^®^, Glaxo Wellcome) 3 MU trice/week for 24 weeks (October 1997–March 1998). In both cases there was no response, neither virological nor serological. During these two courses of therapy, the patient only suffered from minimal and transient side effects. Since then, aspartate aminotransferase (AST) and alanine aminotransferase (ALT) levels were 1.5–2.5 times above the upper limit of normality.

**Figure 1:**
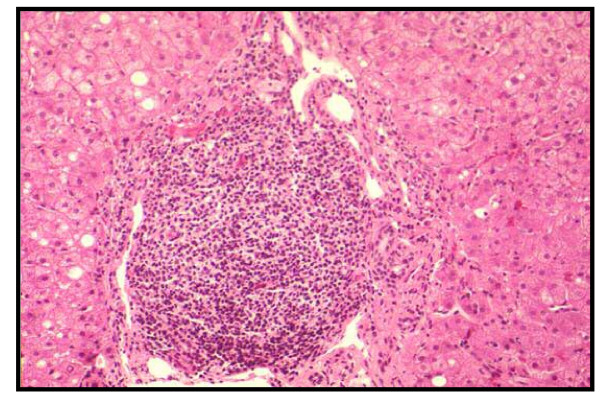
Conspicuous lymphocytic infiltration of portal tracts (Hematoxylin & Eosin, 200 ×).

**Figure 2:**
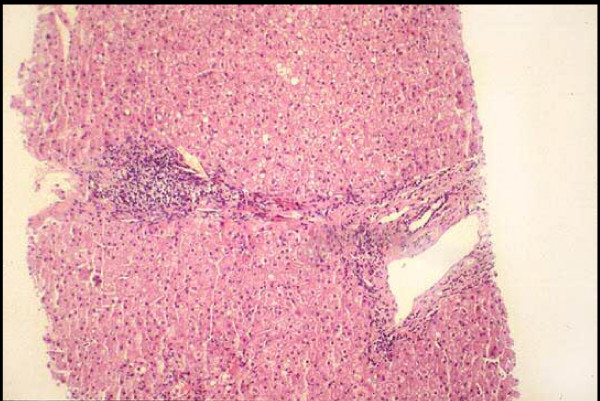
Porto-portal passive septa. Hematoxylin & Eosin, 50 ×.

On admission in October 2001 the AST and ALT levels were 54 and 97 U/L, respectively (normal value ≤ 40 U/l); the platelets count was 179,000 mmc and hemoglobin 13.3 g/dL; HCV-RNA was positive (AxSYMHCV 3.0^® ^Abbott); the viral load was 560,000 IU (Cobas Amplicor HCV Monitor 2.0^® ^Roche); the genotype was characterized as 2a/2c (Genotype HCV III^® ^Nuclear Laser, Milan, Italy). ANA, AMA, SMA, Anti -TPO Ab and Anti-TG Ab were absent; FT3, FT4 and TSH serum concentrations were within the normal range. Liver biopsy, performed in the 1996 and repeated before the treatment, showed a mild hepatitis (Knodell score 13–15/22; Metavir score A2 F2), (Figure 1 and 2). The patient was re-treated with PEG IFN α2b (Peg Intron^®^, Schering-Plough) 100 μg once a week, plus Ribavirin (Rebetol^®^, Schering-Plough) 800 mg/day, for 24 weeks. During the first three doses of Peg IFN the patient suffered from typical self-limited flu-like syndrome, with fever (up to 39°C), arthro-myalgias and asthenia. AST/ALT levels started lowering, i.e., 39/56 U/L and 36/43 U/L at the second and third month, respectively; by the middle of the third month, the HCV-RNA load kept on decreasing until it was more than two LOGs (3,500 IU) at the end of the fourth month of treatment.

**Figure 3:**
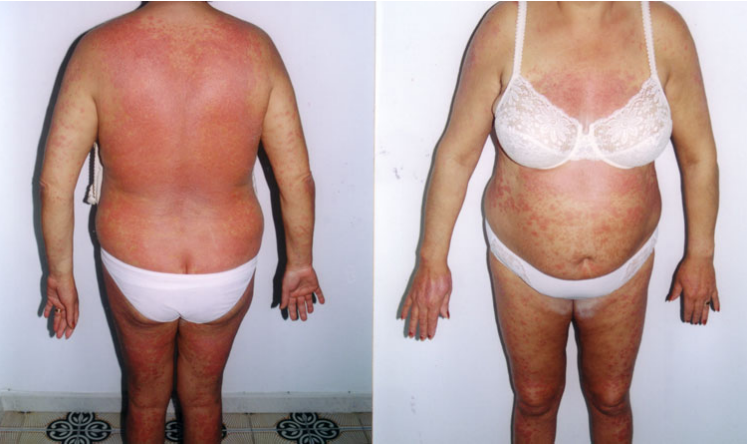
Extensive psoriasis: the body is involved in almost its entirety.

**Figure 4:**
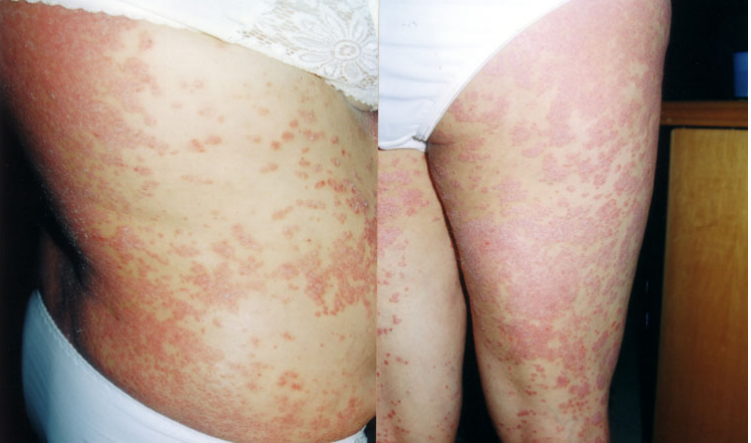
Extensive psoriasis: involvement of trunk and lower limbs.

At the beginning of the third month the patient developed a mild form of plaque psoriasis; this comprised a few, scarcely raised, thickened patches of red skin, covered with silvery-white scales, which were present on the skin surface of the knees, elbows, scalp and trunk, involving less than 10% of the body surface area. The therapy was continued, in accordance with the patient's firm request and based on the encouraging results. The Beck Depression Inventory was performed, without showing evidence of any mood disorders [8]. During the fourth month of treatment, the patient's AST and ALT levels were normalised (23 and 31 U/L, respectively); from then on, these values were always normal. The serum HCV-RNA was negative at the fifth month of therapy; instead, psoriasis worsened, becoming extensive (involving more than 75% of the body surface area) and affecting the thorax, dorsum, abdomen, arms, thighs, and legs (Figure 3 and 4). Joint disease of psoriatic origin (criteria: either greater than two swollen or two tender/painful joints for more than two weeks) did not appear. In any case, the therapy was continued until the sixth month, at which time it was stopped (April 2002), Figure 5.

**Figure 5:**
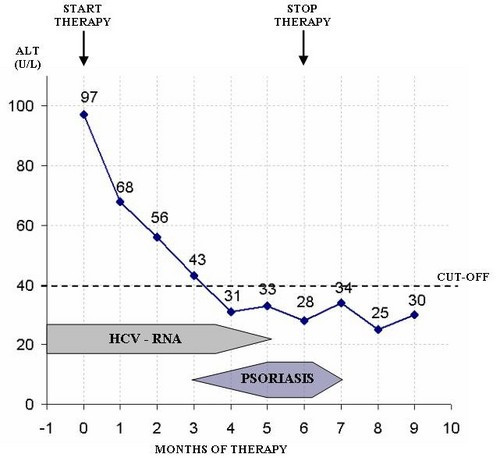
Clinical, laboratory and therapeutical data.

After discontinuation of therapy, the psoriasis spontaneously receded, in a slow but complete fashion, within one month, without any local or systemic therapy. From then on, the patient underwent periodic check-ups which have always showed a sustained response. At the time of publication, and after more than four years of follow-up, the patient has not experienced relapse of either the hepatic disease or the psoriasis.

## Discussion

Extrahepatic manifestations and IFN-induced side-effects sometimes overlap. Mixed cryoglobulinemia is the most studied syndrome associated with this infection. It is a systemic vasculitis that may involve the skin, kidney and nervous system. A frequent association is that between HCV infection and non-Hodgkin lymphoma. Thyroid disease (hypothyroidism) is commonly seen in people with hepatitis C. Other studies describe a correlation between hepatitis C virus and lymphocytic sialoadenitis. Rheumatologic symptoms such as polyarthritis often occur in people with hepatitis C. Finally, hepatitis C infection has been associated with dermatological disorders such as porphyria cutanea tarda and lichen planus. An efficient cure for hepatitis C infection, based on combined antiviral therapy, is available. Side-effects such as flu-like syndrome, depression, haemolytic anemia, cytopenia and alopecia can limit its use.

The patient in this case had received two types of standard IFN in the past, without virological effectiveness, but also without any cutaneous involvement. Therapy with PEG IFN plus Ribavirin led to a sustained response, but also an extensive form of psoriasis. Many clinicians believe that the onset of psoriasis during IFN therapy is an absolute contraindication to its continuation.

In this case the IFN therapy was continued, without any specific intervention for the psoriasis. This was because the AST/ALT levels had improved since the second month therapy, forecasting eradication of HCV especially in the light of a favourable genotype, and spontaneous regression of the cutaneous manifestation was considered possible at the end of therapy cycle once the, trigger that had generated it was withdrawn. The patient wished to complete the therapy (at that time the normal duration of this antiviral therapy combination was six months), as she was very worried about the possible development of cirrhosis and because she was seeing for the first time the levels of AST/ALT diminishing. Moreover, she did not consider the body appearance important. She had no evidence of a mood disorder. This last point played a key role in reinforcing the physicians' decision to continue treatment.

A previous case study reported a 45-year-old woman with chronic hepatitis C who was treated with the same antiviral schedule and who developed psoriasis, after not having experienced symptoms of the condition for the past 10 years. In that case the psoriatic lesions worsened dramatically during therapy. Cutaneous lesions appeared at various sites including the face, the back of the ears, the breasts the anus and the elbows. Because of the severity of the psoriatic disease, therapy was discontinued after 14 weeks from the treatment onset, when the serum RNA was eliminated. The authors reporting that case [9] concluded that this side effect should be kept in mind in the treatment of patients with a history of psoriasis.

In this reported case the therapeutic interruption coincided with viral clearance, but did not answer the question of what is the best approach when viral clearance has not yet been achieved.

Finally, we offer a comment on the pathogenesis of the IFN-induced psoriasis in association with chronic hepatitis C. Psoriasis is considered a T cell-mediated disease, with a strong cytokine component. Whereas pro-inflammatory cytokines such as tumor necrosis factor-alpha is overexpressed in this diseases, a type 1 cytokine pattern predominates. Recently [10] a case has been reported of a patient with psoriasis and hepatitis C virus infection who initially presented with psoriatic erythroderma and eventually showed complete clearance of psoriatic lesions following acute hepatitis induced by etretinate treatment. Cytokine synthesis capabilities in peripheral blood T cells showed a dramatic increase in the frequency of interferon-gamma-producing CD8+ T cells. This process was observed during the erythrodermic stage. In contrast, the frequencies of interleukin (IL)-4- and IL-13-producing CD4+ T and CD8+ T cells were remarkably high at the resolution stage. These results clearly indicate that a shift towards type 2 cytokine predominance contributes to the resolution of severe psoriasis. This interesting observation is in accordance with data indicating that a T-helper (Th) 1 to Th2 shift does not occur in chronic hepatitis C. Further more, IFN alpha alone or in combination with ribavirin acts induces and maintains high rates of significant CD4+ Th 1 response [11].

In conclusion, we acknowledge that no definitive guidelines exist concerning the clinical conduct in this specific situation. Our clinical experience on a single case could contribute to resolving this matter, as appropriate trials are very difficult to implement for ethical reasons.

## Competing interests

The author(s) declare that they have no competing interests.

## Authors' contributions

All the Authors equally participated in the preparation of this case report on the basis of their expertise.

They read and approved the final manuscript.
